# The 3′ end of the story: deciphering combinatorial interactions that control mRNA fate

**DOI:** 10.1186/s13059-017-1360-6

**Published:** 2017-11-29

**Authors:** Jeremy R. Sanford, Luiz O. F. Penalva

**Affiliations:** 1 Molecular, Cellular and Developmental Biology, University of California Santa Cruz, Santa Cruz, CA 95064 USA; 2 Children’s Cancer Research Institute, University of Texas Health Science Center at San Antonio, San Antonio, TX 78229 USA

## Abstract

A new study investigates how microRNAs affect the binding of proteins to RNA.

Precise control of messenger RNA (mRNA) fate—its translation, stability, and localization—is required for accurate eukaryotic gene expression and is of fundamental importance to human health and disease. Initial efforts to determine how the cell controls these processes focused on “who done it?” We now understand that many of the clues required to answer this question reside in the mRNA’s 3′ untranslated region. For example, the *cis*-regulatory elements embedded in the 3′ untranslated region engage *trans*-acting microRNAs (miRNAs) and RNA-binding proteins (RBPs). RBPs and miRNAs package the message into ribonucleoprotein particles (messenger ribonucleoprotein particles, mRNPs) that are remodeled throughout the life cycle of the mRNA. Like transcription factors, these post-transcriptional regulators control the abundance of the message as well as its association with the translation machinery, and therefore dictate the protein output of a gene.

Until recently, attempts to unravel the regulatory impact of RBPs and miRNAs focused on interrogating one of the usual suspects in isolation, using genomic or molecular approaches that provide snapshots of transient interaction sites. Individual and group efforts like ENCODE produced catalogues of these interactions. However, more often than not, these regulatory factors collaborate in unexpected ways to control post-transcriptional gene expression. Therefore, there is a need to develop strategies that allow us to dissect cross-talk among regulators and link mRNA–protein and protein–protein interactions to regulatory outcomes. New work by Rissland and colleagues, published in the current issue of *Genome Biology*, begins pulling the thread connecting combinatorial interactions of miRNAs and RBPs on a global scale [1]. Using a modified RIP (ribonucleoprotein immunoprecipitation) protocol as a genome-wide reporter, they reveal novel regulatory aspects, the dynamics of mRNPs and how the association of miRNAs alters the protein composition of mRNPs.

## The need to combine genomic methods in RNA biology

RIP was initially developed by Jack Keene’s lab in the early 2000s and was the first attempt to put RNA biology in the world of genomics. RIP uses a simple approach where specific mRNPs are isolated via immunoprecipitation and the mRNA component is identified later via microarray or deep sequencing [2, 3]. Although Keene’s inaugural RIP study focused mainly on core translation factors such as PABP and elF4E and changes in their mRNA-associated populations before and after cell perturbations, RIP ended up evolving not as a method to dissect translation or mRNA decay regulation but as a tool to map mRNA targets of specific RBPs. RIP was subsequently replaced by CLIP (cross-linking and immunoprecipitation) developed by Robert Darnell’s lab. Thanks to the use of UV cross-linking to “freeze” RNA–protein interactions, CLIP presents two main advantages: it is a “background-free” method and provides a precise genome-wide binding site map of the RBP under analysis [4]. Later, modified CLIP approaches were developed including some dedicated to the mapping of miRNA sites via the analysis of Ago interactions [5–7]. For many years, most of the RNA genomics related to mRNA decay and translation regulation were restricted to reports describing RBP and miRNA binding site maps. Methods to provide global readings of translation and mRNA decay came much later with Ribo-seq or ribosomal foot-printing and BRIC-seq, respectively [8, 9].

The field is definitely in need of cross-platform studies and novel approaches to expand our knowledge on specific and general mechanisms of translation and mRNA decay regulation. The use of modified RIP protocols combined with other genomic methods as reported by Rissland et al. [1] is an interesting strategy since it allows us to gain mechanistic insight by addressing whether regulators or conditions affect the association of selected core factors to mRNAs and determining the features of the associated mRNA populations.

## Changes in 3′ end

miRNAs regulate mRNA expression by repressing translation and promoting mRNA decay. Although a lot has been learned in past years regarding the mechanism employed by RNA-induced silencing complex (RISC) and other factors involved in miRNA-mediated regulation, there are still many open questions, in particular concerning the sequence of events. Rissland et al. investigated mRNP organization in cell systems by quantifying via RIP the changes in associations between core factors and mRNAs upon miRNA transfection [1].

PABP has been shown previously to be a critical factor in miRNA regulation, functioning initially to recruit the RNA-induced silencing complex to target mRNAs and later being released as part of the degradation step [10]. As expected, PABP occupancy in the mRNA population targeted by the transfected miRNAs was shown to decrease in the presence of the cognate miRNA. Surprisingly, examination of PABP-associated mRNAs showed no differences in poly(A) tail length in control versus miRNA-transfected samples, suggesting that PABP dissociates prior to the deadenylation process. The decay factor DDX6 seems to come after PABP dissociation. miRNA transfections produced a dramatic increase in DDX6 association with target transcripts and those transcripts showed a significant reduction in poly(A) tail length. Perhaps most interesting are results that show novel general aspects of PABP and DDX6 interaction and function. Different from what is commonly believed, poly(A) tail length does not correlate with PABP occupancy and does not correlate with mRNA stability or translation. PABP binding is coordinated with elF4E and elF4G and their occupancy correlates positively with mRNA stability and translation efficiency. In the case of DDX6, the authors suggest that its function goes beyond miRNA-mediated repression. DDX6 associates with a large range of mRNA with short poly(A) tails and, therefore, it could potentially participate in other mRNA-decay pathways.

In summary, the work of Rissland and collaborators shows a new perspective for the use of RIP and other genomic platforms to explore the dynamics of mRNPs and evaluate how miRNAs and RBPs influence mRNA stability, translation, and also RNA processing [1]. It is a major step in RNA genomics that will provide not only a better understanding of basic regulatory mechanisms but also determine how alterations in mRNP composition contribute to diseases and cancer.

## Abbreviations

miRNA: MicroRNA
mRNA: Messenger RNA
mRNP: Messenger ribonucleoprotein particle
RBP: RNA-binding protein
RIP: Ribonucleoprotein immunoprecipitation
